# Estimating the impact of physician risky-prescribing on the network structure underlying physician shared-patient relationships

**DOI:** 10.21203/rs.3.rs-4139630/v1

**Published:** 2024-03-26

**Authors:** Xin Ran, Ellen Meara, Nancy E. Morden, Erika L. Moen, Daniel N. Rockmore, A. James O’Malley

**Affiliations:** 1 Department of Biomedical Data Science, Geisel School of Medicine at Dartmouth, Lebanon, 03756, NH, USA.; 2 The Dartmouth Institute for Health Policy and Clinical Practice, Geisel School of Medicine at Dartmouth, Lebanon, 03756, NH, USA.; 3 Department of Health Policy and Management, Harvard T.H. Chan School of Public Health, Boston, 02115, MA, USA.; 4 National Bureau of Economic Research, Cambridge, 02139, MA, USA.; 5 United HealthCare, Minnetonka, 55343, MN, USA.; 6 Department of Mathematics, Dartmouth College, Hanover, 03755, NH, USA.; 7 Department of Computer Science, Dartmouth College, Hanover, 03755, NH, USA.; 8 The Santa Fe Institute, Santa Fe, 87502, NM, USA.

**Keywords:** Risky prescribing, Shared-patient physician network, Homophily, Deprescribing, Quantifying polypharmacy, State-space, Transition matrix

## Abstract

Social network analysis and shared-patient physician networks have become effective ways of studying physician collaborations. Assortative mixing or “homophily” is the network phenomenon whereby the propensity for similar individuals to form ties is greater than for dissimilar individuals. Motivated by the public health concern of risky-prescribing among older patients in the United States, we develop network models and tests involving novel network measures to study whether there is evidence of geographic homophily in prescribing and deprescribing in the specific shared-patient network of physicians linked to the US state of Ohio in 2014. Evidence of homophily in risky-prescribing would imply that prescribing behaviors help shape physician networks and could inform interventions to reduce risky-prescribing (e.g., should interventions target groups of physicians or select physicians at random). Furthermore, if such effects varied depending on the structural features of a physician’s position in the network (e.g., by whether or not they are involved in cliques – groups of actors that are fully connected to each other – such as closed triangles in the case of three actors), this would further strengthen the case for targeting of select physicians for interventions. Using accompanying Medicare Part D data, we converted patient longitudinal prescription receipts into novel measures of the intensity of each physician’s risky-prescribing. Exponential random graph models were used to simultaneously estimate the importance of homophily in prescribing and deprescribing in the network beyond the characteristics of physician specialty (or other metadata) and network-derived features. In addition, novel network measures were introduced to allow homophily to be characterized in relation to specific triadic (three-actor) structural configurations in the network with associated non-parametric randomization tests to evaluate their statistical significance in the network against the null hypothesis of no such phenomena. We found physician homophily in prescribing and deprescribing in both the state-wide and multiple HRR sub-networks, and that the level of homophily varied across HRRs. We also found that physicians exhibited within-triad homophily in risky-prescribing, with the prevalence of homophilic triads significantly higher than expected by chance absent homophily. These results may explain why communities of prescribers emerge and evolve, helping to justify group-level prescriber interventions. The methodology could be applied to arbitrary shared-patient networks and even more generally to other kinds of network data that underlies other kinds of social phenomena.

## 1 Introduction

Risky prescribing among the older population is a health concern for which public health interventions are critically needed. Risky prescribing commonly refers to the excessive prescribing (“polypharmacy”) of unwarranted prescriptions that deviate from guidelines (Dreischulte and Guthrie, 2012; Gnjidic et al, 2013; Bushardt et al, 2008). In the U.S., the older population consumes more than one-third of prescription medications, yet they consist of around 15% of the population (Fulton and Riley Allen, 2005; Werder and Preskorn, 2003). Even more concerning are the adverse events associated with risky prescribing. For example, evidence was found that the combined usage of opioids and benzodiazepines or non-benzodiazepine sedative-hypnotics (sedative-hypnotics) has a higher risk of overdose than using opioids alone (Cho et al, 2020; for Disease Control et al, 2016; Sun et al, 2017).

Physicians are a major determinant of patient drug regimes, especially for drugs that patients cannot directly access. Social network analysis has proven to be effective for studying collaborations among physicians and their association with patients’ health outcomes (Barnett et al, 2012; Fattore et al, 2009; Pollack et al, 2015; Moen et al, 2016). Understanding how different prescribing behaviors are embedded in the shared-patient physician network may help identify the physicians to intervene on in order for the impact of the intervention to be maximized. For example, the most connected physicians might be the most conducive to having the effects of a behavior change intervention spillover to the greatest number of other physicians. Similarly, if actors with certain shared or similar traits are commonly connected in the network, targeting groups of connected persons with such traits might provide the most effective form of intervention. Assortative mixing or “homophily”, commonly known as “birds of a feather flock together”, is a social phenomenon in which people who share similar traits are more likely to form relationships with each other (McPherson et al, 2001; Apicella et al, 2012; Rand et al, 2011). The existence of homophily can reinforce the influence between a pair of connected individuals in social networks (“dyads”) such that individuals are more prone to interact with individuals they resemble than those they don’t (Centola, 2011).

Previous studies have found that physicians with the same organizational affiliation were more likely to develop professional relationships (Landon et al, 2012; Mascia et al, 2015) and that homophily in a network of opioid users was associated with the number, type and daily dosage of opioid prescriptions (Aroke et al, 2021). These prior works motivate the conjecture that homophily on risky prescribing may generalize to a more complex phenomena that simultaneously across dyads resulting in the emergence of clusters of three or more heavy prescribers. However, with the exception of the preliminary work by Ran et al (2024), to our knowledge structural characteristics of shared-patient physician networks of prescribers of risky drugs such as those in the opioids, benzodiazepines, and sedative-hypnotics classes is understudied.

Previous studies have also documented geographic variation in patient healthcare utilization and outcomes in relation to network characteristics (Landon et al, 2012; Fisher et al, 2003a,b; Wennberg, 2010; Pollack et al, 2012). By comparing physician homophily associated with prescribing within United States (US) states and hospital referral regions (HRR), we additionally investigate whether physician prescribing intensity clusters within and varies across geographic regions, extending the healthcare variations literature in a unique way.

Several studies have quantified patients’ receipt of polypharmacy and physicians’ opioid prescribing patterns among different specialties (Quinn and Shah, 2017; Levy et al, 2015). However, current approaches for quantifying physicians’ prescribing behaviors identify risky prescribing without accounting for the extent to which appropriate deprescribing occurs. Unlike prescribing, deprescribing often takes place in conversations during physician-patient encounters involving reviews of patients’ medications (Farrell and Mangin, 2019) and triggers no insurance claim. This leads to challenges in identifying the physician or physicians responsible for deprescribing. An important contribution of this paper that falls within the emerging field of data science is the development of heuristic algorithms for identifying likely instances of deprescribing in claims data.

Exponential random graph models (ERGMs) provide a general modeling framework for relating network phenomena and actor attributes to the likelihood of observing a network. In theory, they provide an ideal methodological basis for estimating which factors, including those relating to physician prescribing behavior, are most strongly associated with individual network relationships and network structure of the observed network. One challenge with exponential random graph models (ERGMs) is the phenomenon in which the model puts most of its mass on a very dense or sparse network. Known as degeneracy, this phenomenon has been commonly encountered by investigators examining whether dyadic-dependent network phenomena such as transitivity underlie the network (Handcock et al, 2008; Handcock, 2003; Moen et al, 2016). When using ERGMs to study homophily, degeneracy may limit our ability to isolate the true level of homophily from the confounding effects of other network phenomena, such as those embodied in network configurations involving three actors such as transitivity and other forms of triadic dependence. To avoid this problem, we introduce two new network statistics that capture specific triadic phenomena and accompanying statistical tests of whether the prevalence of such phenomena in the network exceeds that expected by chance in the absence of homophily. The two new descriptive statistics quantify risky-prescribing associated homophily acting within closed triadic configurations (“triangles”). One new network statistic is the proportion of closed triangles in which each of the three actors has the attribute in common while the other is the proportion of two-paths (2-stars or open triangles) with the same node attribute that are closed, an attribute-specific version of transitivity (Latapy et al, 2008). We construct statistical tests based on randomly re-distributing the node attributes across the nodes in the network to evaluate the null distribution of the test statistic. Using these new statistics and associated statistical tests, we sought to gain unique insights into the extent to which risky prescribing is associated with the network structure of physician relationships.

The specific contributions of our work include:

- Investigation of prescribing-associated homophily in physician shared-patient networks at the state-level, as well as the variation in homophily across HRR sub-networks.
- A novel framework to quantify physician prescribing behavior by their contribution to patient prescription state transitions and the development of a number of risky prescribing indexes that can be conveniently computed and used as node attributes.
- Heuristic algorithms to attribute physicians to deprescribing prescription events, addressing a current limitation of claims data that physician responsibility for deprescribing is not observable.
- Development of novel triadic network statistics to study prescribing-associated homophily and allied non-parametric statistical tests for determining whether homophily acts beyond dyads, a supra-dyadic effect, partially overcoming the challenge of avoiding degeneracy while testing for triadic dependence in ERGMs.

In the next section, we first introduce the data from which we construct shared-patient physician networks. Then we introduce ERGMs for studying risky-prescribing associated homophily while controlling for network density and the prevalence in the network of node attributes, including deriving the conditions under which the homophily contrasts are linearly independent of other node-attribute terms in ERGMs. We next introduce two descriptive statistics to quantify the triadic homophily of risky prescribing and a non-parametric test for evaluating whether the prevalence of attribute-closed triads in the network exceeds that expected by chance. In describing our motivating application we develop two novel data processing and data science methods for our motivating application: 1) the development of drug-state transition matrices specific to each physician, incorporating both prescribing and deprescribing transitions; 2) the summarization of these matrices in the form of prescribing indexes used as node attributes in a model of the network. Lastly, we describe the results of the risky prescribing study and conclude the paper.

## 2 Network Methods

### 2.1 Overview of network data for the Risky Prescribing Study

**Fig. 1:**
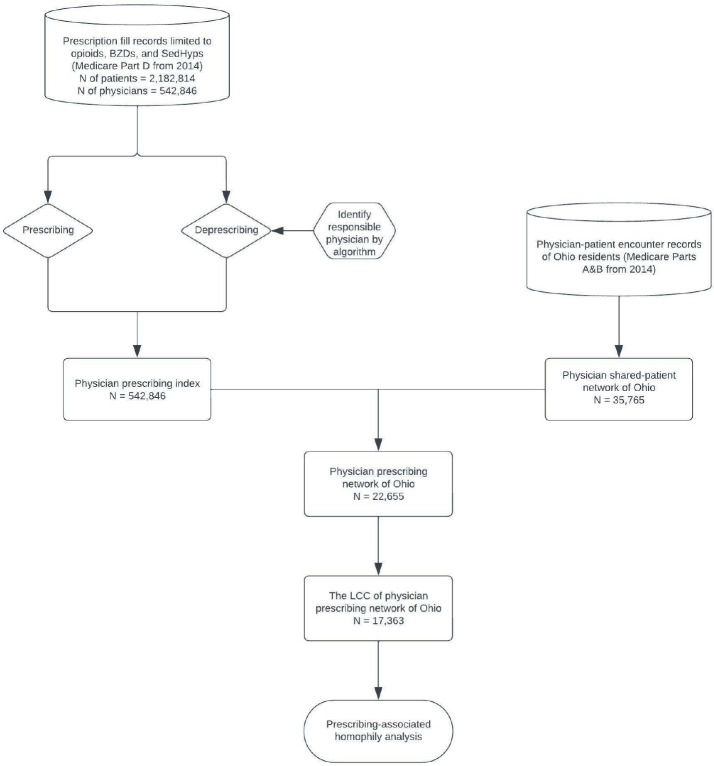
Study cohort definition and workflow. Note: Beneficiaries were included in the study if they had at least 3 months of continuous coverage of Medicare Parts A, B, and D, and 2 years of continuous parts A and B coverage prior to cohort entry. LCC = largest connected component.

We used a 40% random sample of all Medicare fee-for-service claims in 2014 of beneficiaries residing in the state of Ohio to extract relevant physician-patient encounters for constructing the physician network. Because the complete physician-patient encounter data was only available for patients residing in the state of Ohio, we limited our shared-patient physician network to physicians caring for Ohio residents in 2014. The formation of the shared patient physician network is one half of the two intersecting datasets developed to enable the analysis of the motivating application; the other is described in Section 3.1 and Figure 1, which outlines the risky prescribing study data and data wrangling procedures.

### 2.2 Shared-patient physician network

A unipartite physician network is constructed from the ensemble of physician-patient encounters observed in Medicare fee-for-service claims data in 2014. A visit to physician i followed by a visit to physician j by the same patient within 2014 is considered to provide evidence of a meaningful professional relationship(“patient referral”) from physician i to j (O’Malley et al, 2022; An et al, 2018b,a). Because a dyad with a bidirectional relationship is most likely to involve physicians who have made a deliberate choice to refer their patients to one another, we restricted the connections between physicians to mutual ties (those dyads with edges in both directions). We transformed the network to an undirected binary network using a threshold of 0, which amounts to requiring that at least one patient encounters one physician first and then the other and vice-versa for at least one other patient. We limit the network to physicians who had at least prescribed one drug in the drug classes of interest, including opioids, benzodiazepines, or sedative-hypnotics in 2014 according to Medicare Part D data. Finally, we reduce the network to its largest connected component (LCC) to eliminate isolated dyads (pairs of physicians who only shared patients with each other and thus have a network degree of 1) as such physicians were likely practicing in a part-time or other reduced manner.

To study variation in homophily across HRRs and the possible variation of risky-prescribing-associated homophily across different HRRs, we divided the LCC of the physician prescribing network into HRR sub-networks according to where the majority of their patients reside based on their Medicare fee-for-service claims in 2014. Both physicians must belong to the same HRR in order for the edge between them to be in that HRR sub-network.

### 2.3 Exponential random graph models (ERGMs)

An ERGM is an exponential family model for relational data. Standard regression models cannot handle sociocentric network data if the status of the edges (ties) in the network are statistically dependent as this violates the independence and no-interference assumptions of standard regression (Contractor et al, 2006). ERGMs overcome this issue and allow nodal attributes, edge attributes, dyadic dependencies, and some higher-order network dependencies to be simultaneously modelled (Robins et al, 2007a,b; Snijders et al, 2006). ERGMs model the probability distribution of all possible networks given a set of nodes, a discrete-value distribution of a categorical random variable with a large number of possible states (O’Malley and Marsden, 2008), and in estimation seek the parameters weighing the importance of each network statistic that make the observed network the most likely among all of its possible realizations (Robins et al, 2007a). Mathematically, ERGMs model the probability that a random network A is realized by an observed network $a\;(a_{ij}=1$ if there is an edge between node i and j and 0 otherwise) as:

(1) $$Pr(A=a|X=x)=\frac{1}{\kappa (x)}exp\left\{\sum_{p}\;\eta_{p}g_{p}(a,x)\right\}$$

where $\kappa (x)=\sum_{a\in 𝒜}\;exp\{\sum_{p}\;\eta_{p}g_{p}(a,x)\}$ is a normalizing constant that makes the probabilities sum to 1 across possible networks with the given number of nodes and having attributes X with realized value x (Goodreau, 2007; O’Malley and Marsden, 2008); the vector of attributes of node i is the *i*th row of x, denoted $x_{i}^{T}$; and $g_{p}(a,x)$ is the *p*th network statistic. A positive $\eta_{p}$ for the network configuration represented by $g_{p}(a,x)$ indicates that the model for the network favors networks with feature p while a negative value indicates that networks with a high prevalence of feature p have lower likelihood. The parameter $\eta_{p}$ quantifies the change in the conditional log odds of a tie under a one-unit increase in network statistic p holding the other terms in the model fixed, which often isn’t possible given the common dependence of the network statistics on **a**. Commonly used network statistics include the number of edges, the number of reciprocated or mutual edges (for directed networks), certain degree-related configurations (e.g., k-stars), triadic configurations (e.g., triangles, transitive triads, three-cycles), and nodal or edge-level attributes (e.g., node factor and node covariates). The ergm package in R contains an extensive list of network statistics (Hunter et al, 2008).

In theory, any network statistic may be included as a predictor in (1) and in practice a wide range of statistics capturing various network features have been represented in ERGMs (Morris et al, 2008). The term for the number of edges accounts for the density of the network and so is often included. Herein, network statistics that quantify the level of homophily of specified attributes in the network are of primary interest.

### 2.4 Homophily statistics: Isolating their effect and associated identifiability results

Homophily can be thought of as a within-dyad interaction between the two nodes comprising the dyad of the given attribute (Hunter et al, 2008; Morris et al, 2008). When studying the homophily of an attribute, it is important to adjust for the main effect of the node attribute to ensure that homophily is a relative measure as opposed to being confounded by the prevalence of the attribute across the network. The network statistics associated with these main effects are often named *nodecov* (for continuous attributes) and *nodefactor* (for categorical attributes) in ERGMs. Table 1 shows the mathematical definitions of the ERGM terms used in this study and their interpretations. When estimating homophily while adjusting for the node-level effect of the same attributes, only the effect of uniform homophily (the effect of having the same value of the attribute irrespective of its value) can be estimated. In contrast, under differential homophily, the coefficients of the network statistics are unidentifiable due to linear dependencies between the predictors. For illustration, suppose that $x_{i}$ is a scalar (i.e., each node in the network has a single attribute). For a binary node attribute x taking a value of 0 or 1, the network statistic for the *nodefactor* term is given by,

(2) $$\frac{1}{2}\sum_{ij}\left(x_{i}+x_{j}\right)a_{ij}.$$

The two statistics added by the differential homophily *nodematch* term are,

(3) $$\frac{1}{2}\sum_{ij}x_{i}x_{j}a_{ij}$$

for the node attribute taking a value of 1, and

(4) $$\frac{1}{2}\sum_{ij}\left(1-x_{i}\right)\left(1-x_{j}\right)a_{ij}=\frac{1}{2}\sum_{ij}\left(1-\left(x_{i}+x_{j}\right)+x_{i}x_{j}\right)a_{ij}$$

for the node attribute taking a value of 0 (Table 1). For a binary node attribute, the three predictors in Equations (2), (3), and (4) are linearly dependent and an ERGM for an undirected network including the *edges* term, the *nodefactor* term, and two *nodematch* terms of differential network homophily statistics is not identifiable. The lack of identifiability is seen by the fact that the *nodematch* term (Equation 4) is the sum of the *edges* (Table 1), *nodefactor* (Equation 2), and the 1-level *nodematch* (Equation 3) terms. Therefore, when controlling for the network density with the *edges* term and the main effect of an attribute with the *nodefactor* term, the uniform homophily statistic $\frac{1}{2}\sum_{ij}\;\left(\left(1-x_{i}\right)\left(1-x_{j}\right)+x_{i}x_{j}\right)a_{ij}$ (the sum of the two *nodematch* terms) can be identified but its components (the two differential homophily terms) cannot.

We focus on the homophily of prescribing behaviors and homophily of physician specialty in the risky-prescribing-specific network. We added the uniform homophily terms involving the prescribing indexes one at a time to the model, adjusting for network density, the differential effect of physician specialty on network density (i.e., the physician specialty *nodefactor* term), and the uniform homophily of physician specialty. The R package statnet was used to estimate the resulting ERGM (Hunter et al, 2008; Handcock et al, 2008).

### 2.5 New network statistics: Triadic homophily associated with risky prescribing

Models including only the network statistics discussed to date are dyadic independent and can be estimated straightforwardly. However, as intuited from the fact that the status of one of the three triads comprising a tetrad restricts the possible status of the triad with which it shares an edge, network statistics for triadic terms constrain the parameter-space of an ERGM and induce statistical dependencies in estimation. To avoid these issues along with model degeneracy in estimating an ERGM, we computed two triadic statistics that are restricted through the involvement of attribute information:

- The proportion of closed triangles with the same node attribute, ${Tri}_{1}(a,x)$.
- The proportion of open two-paths (2-stars or open-triangles) with the same node attribute that are closed, $Tri_{2}(a,\;x)$, where $A=\left[a_{ij}\right]$ is the adjacency matrix of the binary-undirected network (see Section 2.2) such that $a_{ij}=1$ if physician i and j shared at least one patient during 2014.

For a binary node attribute (taking the value of 0 or 1), the statistic $Tri_{1}(a,x)$ is defined as,

(5) $${\mathrm{Tri}}_{1}\left(a,x\right)=\frac{\sum x_{i}x_{j}x_{k}\;\cdot \;a_{ij}a_{jk}a_{ki}}{\sum a_{ij}a_{jk}a_{ki}}.$$

Likewise, the statistic $Tri_{2}$ is defined as,

(6) $${Tri}_{2}(a,x)=\frac{\sum x_{i}x_{j}x_{k}\;\cdot \;a_{ij}a_{jk}a_{ki}}{\Sigma x_{i}x_{j}x_{k}\;\cdot \;a_{ij}a_{ik}}.$$

**Fig. 2:**
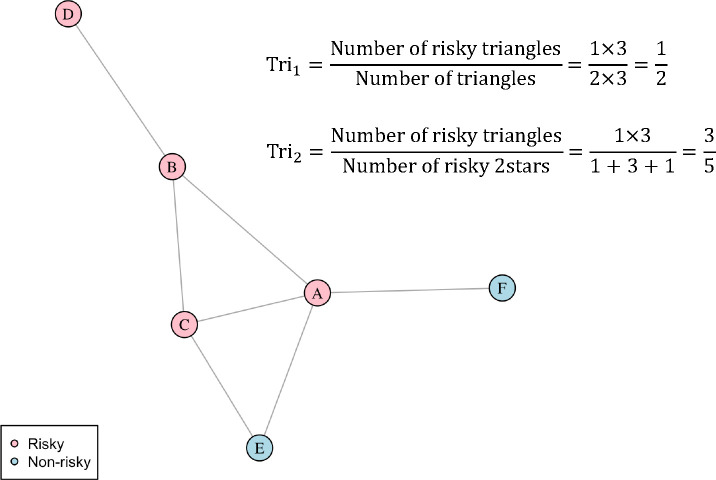
Illustrative computation of triadic homophily statistics ${Tri}_{1}(a,x)$ and ${Tri}_{2}(a,x)$. Suppose nodes A, B, C, and D are physicians who have contributed to risky prescribing, and nodes E and F are non-risky-prescribing physicians. The number of risky 2-stars with nodes A, B, C, and D being the center vertex is 1, 3, 1, and 0, respectively. Therefore, the total number of 2-stars among risky prescribing physicians is five.

Thus, ${Tri}_{1}(a,x)$ is the proportion of times that three physicians who shared patients among themselves all contributed to risky prescribing, while ${Tri}_{2}(a,x)$ is the proportion of triads in the 2-star with physician i as the apex (e.g., the undirected 2-path from j to k via i) that are closed (physicians j and k also shared patients) among those for which nodes i, j, and k are all risky prescribers. Therefore, ${Tri}_{2}\left(a,x\right)\;$ is an attribute-restricted version of node transitivity (Latapy et al, 2008). See Figure 2 for an illustrative example of ${Tri}_{2}(a,x)$ and ${Tri}_{2}(a,x)$.

### 2.6 Non-parametric test for triadic homophily

The numerator and denominator in (5) have their respective ERGM terms in the statnet package (Handcock et al, 2008). However, the ratio of them does not. Similarly, the denominator in (6), the total number of 2-stars among nodes with a certain attribute, is a statistic that is not directly available in statnet. Instead, we perform non-parametric tests by randomly re-distributing the node attribute in question across the nodes, preserving the total number of nodes, the number of nodes with a certain attribute, and the structure of the observed network. We performed 30 random permutations of the attribute of interest across the nodes of the network. We then computed $Tri_{1}(a,x)$ and ${Tri}_{2}(a,x)$ in each of the 30 attribute permuted networks and used the resulting empirical distribution of the test statistic to evaluate the plausibility of the observed value of the statistic under the null hypothesis of no homophily. $Tri_{1}$ and $Tri_{2}$ generalize to continuous node attributes by standardizing the attribute to have a range from 0 and 1. All the analyses were performed using Python 3.7 and R (R Core Team, 2022; Van Rossum and Drake, 2009).

## 3 Measures of homophily in physician prescribing and deprescribing

In this section, we construct measures of physician risky prescribing to be node attributes in ERGMs of the form of (1) to study the homophily of risky prescribing in physician shared-patient networks.

### 3.1 Overview of prescription drug data for the Risky Prescribing Study

A 40% random sample of Medicare Part D claims (prescription drug events) from 2014 was used to retrieve beneficiaries’ prescription fill records and their corresponding prescribers for three classes of risky drugs: opioids (O), benzodiazepines (B), and sedative-hypnotics (S). The beneficiaries’ prescription records, including the physicians who prescribed their drugs from Medicare Part D claims, were used to trace the trajectories of patients’ prescriptions and to construct physicians’ prescribing indexes (see Figure 1 for map of study data formation).

### 3.2 Data preprocessing

To quantify physicians’ prescribing behaviors, our primary interest is in new prescription fills instead of refills because they represent a definitive step towards increased polypharmacy or risky drug combinations. To help distinguish the two, we implemented an empirical rule of 20% overlapping fill length, where a subsequent prescription fill of the same drug written by the same physician was merged to the preceding fill if they overlapped or the gap in between was less than 20% of the fill length of the preceding prescription. It is also highly likely that a subsequent prescription of the same drug signed by a different physician that overlaps with this 20% buffer zone is still a refill of the preceding prescription. Therefore, subsequent prescription fills satisfying the 20% buffer were joined to the preceding fill and attributed to the initializing physician. Such preprocessing enabled us to reduce false positive discontinuations by distinguishing the discontinuation of a prescription from a temporary stop prior to a refill.

### 3.3 Modeling patient prescription states

**Fig. 3:**
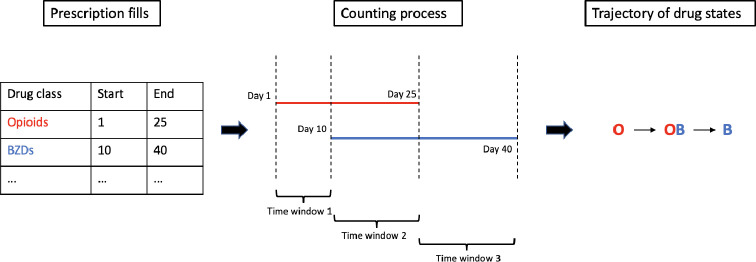
Workflow of modeling patient prescription states. Note: The left-hand panel (L) shows a made-up example of a patient’s sequence of prescription fills with their corresponding drug class. The center panel (C) shows the counting process to split the sequence of prescription fills into discrete exposure time intervals that reflect the initialization and the discontinuation of a prescription fill. The red line indicates the prescription fill length of the opioid in panel (L), and the blue line indicates the benzodiazepine (BZD) fill length. The right-hand panel (R) shows the corresponding prescription state during each time interval in panel (C) and the transition between them, forming a trajectory of prescription states across time. “O” stands for filling an opioid, “B” stands for filling a BZD, and “OB” stands for filling an opioid and a BZD concurrently.

For each of the patients, their prescription fills of the three-drug classes of interest were divided into discrete time intervals, capturing the initialization and discontinuation of a prescription, with each time interval reflecting the number and class of drugs they were exposed to. Based on the number and the class of drugs a patient was taking during each of the discrete time intervals, we assigned each patient prescription state interval to one of the 2^3^ = 8 combinations of prescription states; see Figure 3 for the workflow of modeling patient prescription states. Every initialization or discontinuation of a drug will lead to the changing of a patient’s prescription state. State *zero* is a state of taking no drugs in the three-drug classes of interest. States {O,B,S} correspond to taking at least one drug in precisely one of the opioids, benzodiazepines, or sedative hypnotics drug classes. States {OB,OS,BS} correspond to taking drugs in at least two different drug classes concurrently. State OBS indicates concurrent receipt of at least one drug in each of three different classes. For the ease of mathematical notation in the following sections, 1 to 8 were assigned to the eight prescription states. A string of numbers from 1 to 8 reflecting the sequence of prescription states a patient was in, including both prescribing and deprescribing events, was thereby obtained for each patient. Whereas we consider a patient taking drugs in multiple drug-groups as in a riskier state than a patient taking only one, the drug-groups are not themselves ordered based on severity. That is, herein we consider a lone benzodiazepine to have equal risk as a lone opioid, and the combination of a benzodiazepine and an opioid to have equal risk as the combination of a sedative-hypnotics and an opioid but any combination of two drug states (states 5, 6 and 7) is considered more risky than any of the three singleton drug states (2, 3, and 4).

### 3.4 Attributing physician responsibility to prescribing and deprescribing prescriptions

We attributed the physician(s) who initiated the prescription according to Medicare Part D claims data as the responsible physician(s). For example, the physician who prescribed an opioid to a patient who was already taking a benzodiazepine is the one responsible for the patient’s prescription state transition from state B to state OB (or from state 3 to state 5 using numerical notation). However, due to challenges in identifying the physician or physicians responsible, current approaches for analyzing claims data do not account for the extent to which appropriate deprescribing occurs. Although deprescribing often takes place in conversations during physician-patient encounters where the physician reviews and discusses medications with their patients (Farrell and Mangin, 2019), unlike prescribing it does not trigger an insurance claim. Therefore, there is no record of a responsible deprescribing physician in the Medicare data leading us to develop heuristic algorithms to fill this gap by identifying likely instances of deprescribing in claims data. In developing our deprescribing algorithms, we assume that a deprescribing conversation took place during the patient’s most recent clinical visit if they no longer refill a long-term prescription following their most recent physician encounter (Algorithms 1 and 2 in the Appendix). Briefly, each prescription of each patient is initially treated as a target prescription that can potentially be discontinued. We then sought to exclude the prescriptions for acute conditions to obtain a set of prescriptions for which intentional deprescribing by physicians could have occurred by requiring the target prescriptions to be longer than 30 days. The start date of a prescription fill is assumed to be the date when the patient visited the physician. The physician that the patient visited most recently before the end of the target prescription is selected as the candidate physician for having deprescribed the target prescription. The patient has to discontinue filling the prescription within 30 days (inclusive) after visiting the candidate physician. Recall that any subsequent prescriptions are joined with the preceding prescription if they are likely to be refilled as described in Section 3.1. This data wrangling step is preprocessing procedure that protects against any temporary suspension of a prescription that a patient may refill later on, which can be a false positive deprescribing event by the identified physician. Finally, the patient prescription state transition associated with such termination was attributed to the identified physicians with a contribution weight if multiple responsible physicians were identified. In this way, we obtained as balanced information regarding each physician’s prescribing and deprescribing activities as possible.

### 3.5 Physician transition responsibility matrix

Given the 8 different states a patient can be in at any given time, an 8 by 8 transition responsibility matrix was established for every physician to capture their contribution to prescribing and deprescribing reflecting in their patients’ prescription state transitions.

The physician transition responsibility count matrix (PTRCM) is constructed for each physician by summarizing across all the patients to whom they’ve prescribed and deprescribed drugs in the three classes. The rows of PTRCM correspond to patients’ prescription states in the preceding exposure time interval, while the columns correspond to patients’ prescription states in the current time intervals. For physician k, the PTRCM is given by

(7) $${PTRCM}^{\left(k\right)}=\left[\begin{array}{cccccc} C_{1,1}^{\left(k\right)} & C_{1,2}^{\left(k\right)} & \ldots & C_{1,j}^{\left(k\right)} & \ldots & C_{1,8}^{\left(k\right)}\\ \vdots & \vdots & \ddots & \vdots & \ddots & \vdots \\ C_{i,1}^{\left(k\right)} & C_{i,2}^{\left(k\right)} & \ldots & C_{i,j}^{\left(k\right)} & \ldots & C_{i,8}^{\left(k\right)}\\ \vdots & \vdots & \ddots & \vdots & \ddots & \vdots \\ C_{8,1}^{\left(k\right)} & C_{8,2}^{\left(k\right)} & \ldots & C_{8,j}^{\left(k\right)} & \ldots & C_{8,8}^{\left(k\right)} \end{array}\right].$$

where $C_{i,j}^{\left(k\right)}$ is the total number of patient prescription state transitions from (prescription) state i to state j for which physician k was deemed responsible. To mathematically depict the calculation of $C_{i,j}^{\left(k\right)}$, let h denote a patient, s the prescription change occasion, $D_{hs}$ patient h’s prescription state after prescription change s, and $P_{hs}$ the corresponding responsible physician. Then

(8) $$C_{i,j}^{\left(k\right)}=\sum_{h}\sum_{s}I\left(D_{hs}=i,D_{h\left(s+1\right)}=j\right)I\left(P_{hs}=k\right).$$

where I(event)=1 if event is true and 0 otherwise. To account for the scenario when multiple physicians are responsible for a prescription state transition from i to j, suppose the total number of responsible physicians for prescription state transition s of patient h is $N_{hs}$ and then compute

(9) $$C_{i,j}^{\left(k\right)}=\sum_{h}\sum_{s}I\left(D_{hs}=i,D_{h\left(s+1\right)}=j\right)\frac{\sum_{r}I\left(P_{hsr}=k\right)}{N_{hs}}.$$

### 3.6 Quantitative measures of physician prescribing behavior

#### 3.6.1 Difference between prescribing and deprescribing

We define summary measures of the PTRCM as prescribing indexes corresponding to various aspects of decision-making during physicians’ prescribing practice. First, we compute the relative difference between the number of prescribing and deprescribing events. The elements above the diagonal of the PTRCM contain all of the prescribing transitions while the elements below the diagonal are the deprescribing transitions. By construction, transitions between the same prescription states (these were subsumed by the continuation of the prior fill event as described in the Materials section). The resulting measure for physician k is given mathematically as,

(10) $$I_{\mathrm{base}}^{\left(k\right)}=\frac{\sum_{j}\;\sum_{i<j}\;C_{i,j}^{\left(k\right)}-\sum_{i}\;\sum_{i>j}\;C_{i,j}^{\left(k\right)}}{\sum_{j}\;\sum_{i<j}\;C_{i,j}^{\left(k\right)}+\sum_{i}\;\sum_{i>j}\;C_{i,j}^{\left(k\right)}}.$$

A second family of measures is instead based on the total number of drugs changed (not the number of transitions) to which the physician contributed, obtained by multiplying the count of transitions with the number of drug changes involved. For example, a transition from state B (or state 3) to state OBS (or state 8) involves a change of two drugs, as does the transition in the opposite direction. Let $I_{num}(\omega )$ quantify the number of drugs a patient is taking under the prescription state ω, given by,

(11) $$1_{\mathrm{num}}(\omega )=\left\{\begin{array}{ll} 0, & \omega =1\\ 1, & \omega \in \left\{2,\;3,\;4\right\},\\ 2, & \omega \in \left\{5,\;6,\;7\right\},\\ 3, & \omega =8. \end{array}\right.$$

The resulting risky prescribing index of physician k is defined as,

(12) $$I_{\alpha }^{\left(k\right)}=\frac{\sum_{j}\sum_{i<j}C_{i,j}^{\left(k\right)}{\left|I_{num}(i)-I_{num}(j)\right|}^{\alpha }-\sum_{i}\sum_{i>j}C_{i,j}^{\left(k\right)}{\left|I_{num}(i)-I_{num}(j)\right|}^{\alpha }}{\sum_{j}\sum_{i<j}C_{i,j}^{\left(k\right)}{\left|I_{\mathrm{num}\;}(i)-I_{\mathrm{num}\;}(j)\right|}^{\alpha }+\sum_{i}\sum_{i>j}C_{i,j}^{\left(k\right)}{\left|I_{\mathrm{num}\;}(i)-I_{\mathrm{num}\;}(j)\right|}^{\alpha }}.$$

Here α takes the value of 0 or 1 to define two measures distinguished by whether they account for the number of drug changes involved in the prescription state transition from state i to j. The index $I_{\alpha }^{\left(k\right)}$ is bounded between −1 (exclusive) and 1 (inclusive). The index $I_{\mathrm{base}}^{\left(k\right)}$ defined in (10) is the special case of $I_{\alpha }^{\left(k\right)}$ in (12) when α=0.

#### 3.6.2 Prescribing involving risky prescription state

Our next measure quantifies the extent of physicians’ involvement with the riskiest form of prescribing, state OBS (or state 8), given mathematically as,

(13) $$I_{OBS}^{\left(k\right)}=\frac{\sum_{i<8}C_{i,8}^{\left(k\right)}}{\sum_{j}\sum_{i<j}C_{i,j}^{\left(k\right)}+\sum_{i}\sum_{i>j}C_{i,j}^{\left(k\right)}}$$

where $C_{i,8}^{\left(k\right)}$ is the number of transitions physician k contributed to for which patients transitioned from state i to state 8 (state OBS). We construct another measure by binarizing the numerator of (13) to obtain an indicator of whether physician k has ever contributed to bringing a patient into state OBS:

(14) $$I_{everOBS}^{\left(k\right)}=I\left(\sum_{i<8}C_{i,8}^{\left(k\right)}>0\right).$$

#### 3.6.3 Quantifying extent of prescribing and deprescribing

We also developed several measures to quantify the intensities of physicians’ prescribing and deprescribing. First, we quantified the percentage of patients’ prescription drug state transitions a physician contributed to that involved two or more drug changes. For example, a transition from state *zero* to state OBS, and a transition from state OBS to state B involves three and two drug changes respectively, the former by prescribing and the latter by deprescribing. For physician k, the proportion of their prescribing that involves drug changes in two or more of the three targeted drug classes is given by

(15) $$I_{\mathrm{presc}2mr}^{\left(k\right)}=\frac{\sum_{j}\sum_{i<j}C_{i,j}^{\left(k\right)}I\left(\left|I_{\mathrm{num}}(i)-I_{num}(j)\right|\geq 2\right)}{\sum_{j}\sum_{i<j}C_{i,j}^{\left(k\right)}}.$$

Likewise, the proportion of physician k’s deprescribing that involves changing the status of two or more drug classes is,

(16) $$I_{\mathrm{depresc}2mr}^{\left(k\right)}=\left\{\begin{array}{ll} \frac{\sum_{i}\sum_{i>j}C_{i,j}^{\left(k\right)}I\left(\left|I_{num}(i)-I_{num}(j)\right|\geq 2\right)}{\sum_{i}\sum_{i>j}C_{i,j}^{\left(k\right)}} & ,\sum_{i}\sum_{i>j}C_{i,j}^{\left(k\right)}\neq 0,\\ 0 & ,\sum_{i}\sum_{i>j}C_{i,j}^{\left(k\right)}=0. \end{array}\right.$$

The above measures may enter an ERGM as categorical or continuous node attributes.

## 4 Results: Homophily of risky prescribing as a determinant of physician networks

### 4.1 Physician shared-patient networks

The Ohio shared-patient physician network constructed for this study consists of 35,765 physicians who had clinical encounters with patients residing in Ohio in 2014. The edges in the network reflect the presence of shared patient visits in both directions within the corresponding physician dyad, identified from the sequence of patient-physician encounters observed in Medicare fee-for-service data. After linking physicians in this Ohio shared-patient network to their prescribing measures identified from Medicare Part D data, 22,655 physicians were included in the Ohio shared-patient prescribing network for the three classes of risky drugs. The largest connected component (LCC) of the Ohio shared-patient prescribing network contains 17,363 physicians (see Figure 1). For the HRR sub-network analyses, only the 12 HRR sub-networks with at least 100 physicians, the smallest network size for which prescribing behavior could be measured stably for all physicians in the network, were retained.

Table 2 shows the network statistics of the entire Ohio shared-patient physician network, the prescribing network, and the LCC of the prescribing network. Around 63% of physicians in the Ohio shared-patient physician network were identified as prescribers of at least one opioid, benzodiazepine, or sedative-hypnotic, and around half of the ties in the network took place among the prescribers. The LCC of the prescribing network consists of more than 76% of physicians and more than 98% of the ties. The prescribing network and its LCC were similar in terms of network statistics and physician prescribing measures, except that physicians in the LCC had a slightly higher average node degree (just above 30) and number of Ohio patients encountered annually (nearly 92 patients on average). The facts that the average degree and average volume of patients are both higher within the LCC of the network makes intuitive sense as it is reasonable to expect the restriction to the LCC is akin to selecting more higher connected physicians who see more patients.

### 4.2 Prescribing and deprescribing measures

We first present the network statistics of each of the entire Ohio shared-patient physician network, the prescribing network, and the LCC of the prescribing network. In the LCC of the Ohio physician prescribing network, the distributions of both indexes based on the difference between prescribing and deprescribing; i.e., $I_{0}$ and $I_{1}$, are skewed to the left (Table 2). On average, among all the patient prescription state transitions a physician contributed to, around 0.9% of them involve bringing patients to the riskiest state OBS. Around 8.9% of physicians have at least once contributed to a patient’s transition to state OBS. Among all of the transitions associated with prescribing, around 2.9% of them involved adding two or more drugs. Likewise, among all the transitions associated with deprescribing, on average around 1.6% involved a reduction of two or more drugs. These proportions are nearly invariant between the prescribing network and its LCC.

**Fig. 4:**
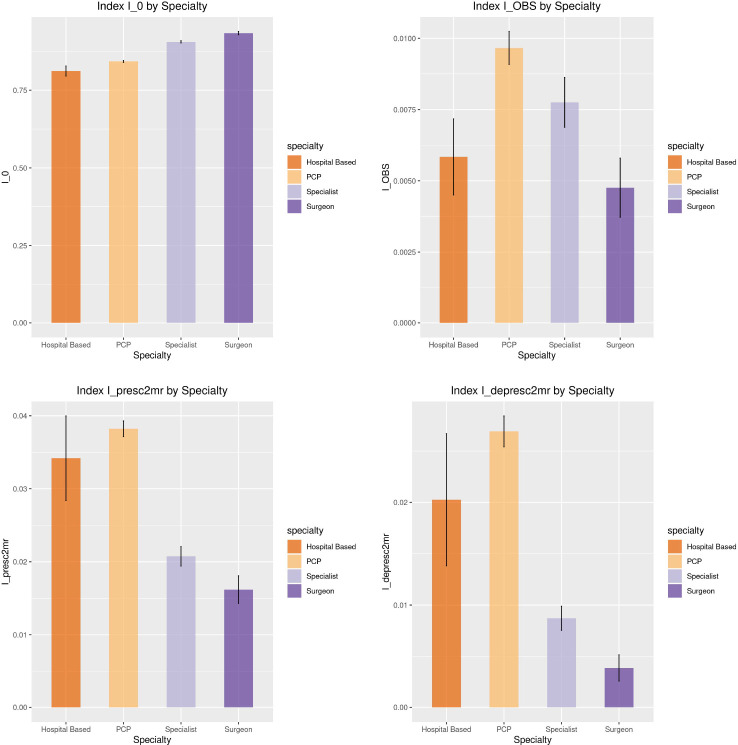
Prescribing measures by specialty of physicians in the largest connected component of the shared-patient prescribing physician Ohio network in 2014. Specialists are medical specialists other than surgeons. Hospital-based services include anesthesiology, radiology, and pathology. PCP denotes primary care physicians. $I_{OBS}$ is the prescribing index based on a physician’s contribution to bringing patients to prescription state OBS, $I_{\mathrm{presc}2mr}\left(I_{\mathrm{depresc2mr}}\right)\;$ is the prescribing index based on a physician’s contribution to prescribing (deprescribing) two or more drug types to patients. Error bars show the standard errors of the respective measures.

Figure 4 presents mean values of four prescribing indexes by physician specialty for the LCC of the shared-patient prescribing physician network of Ohio in 2014; each physician was classed as either a primary care physician, medical specialist, and surgeon specialist based on their lookup information in the National Plan and Provider Enumeration System (NPPES) (Centers for Medicare & Medicaid Services, 2012). In terms of overall prescribing and deprescribing reflected by $I_{0}$, there was minimal difference across specialties, although surgeons and medical specialists appeared to have slightly higher average $I_{0}$ values than other specialties. Other prescribing measures reflect physicians’ prescribing behavior with more granularity. Hospital-based physicians (often referred to as hospitalists) and primary care physicians (PCPs), in particular, have a higher likelihood of bringing patients to state OBS, prescribing two or more drugs, and deprescribing two or more drugs, compared to medical specialists and surgeons. While the relative magnitude of these differences is large, the absolute magnitude is modest as the three types of transitions at the core of these measures are at best infrequent and in the case of $I_{OBS}$ are rare occurrences. The confidence interval for hospitalists is the widest, implying that such physicians are the least common.

### 4.3 ERGMs for adjusted homophily

Table 3 shows the estimated ERGM-adjusted homophily effects in the LCC of the shared-patient prescribing physician network. When controlling for network density and the main effects of nodal prescribing and deprescribing attributes, the network exhibited assortative clustering in terms of different prescribing measures. An overall homophily effect was found among physicians in ever bringing patients to the OBS state (est.=0.037,p<0.001). The status of connections among physicians were associated with the continuous prescribing measures; because these are distance measures, a negative coefficient estimate implies greater homophily. Physicians with a larger difference in their likelihood of transitioning patients to OBS were less likely to be connected to each other (est.=-1.200,p<0.001) while those with larger differences in the likelihood of prescribing two or more drugs to patients at once had a lower likelihood of a tie (est.=-0.619,p<0.001). Similarly, ties were less likely if there was a larger difference in their propensity to deprescribe two or more drugs, although the effect is substantially lower in magnitude (est.=-0.203,p<0.01). The estimated effect of physicians’ propensity to form ties with other physicians of the same specialty was consistent across all models. Compared to PCPs, emergency medicine physicians, neurologists, and psychiatrists had fewer connections with other physicians in the network. After controlling for the main effect of physician specialty, PCPs and neurologists were less likely to be connected to same specialty physicians. In contrast, emergency medicine physicians and psychiatrists were more likely to form ties with physicians of the same specialty. We believe the latter finding reveals that psychiatrists are more likely to send a patient to another psychiatrist for a second opinion or that patients are more likely to doctor-shop among psychiatrists than among other specialists and especially PCPs. In contrast, PCPs seldom refer patients to other PCPs. Finally, the positive main-effects of the prescribing indices and the negative main-effects for Speciality reflect that physicians involved in more prescription transitions have more ties in the network and that PCPs have more network ties to other physicians than do specialists.

**Fig. 5:**
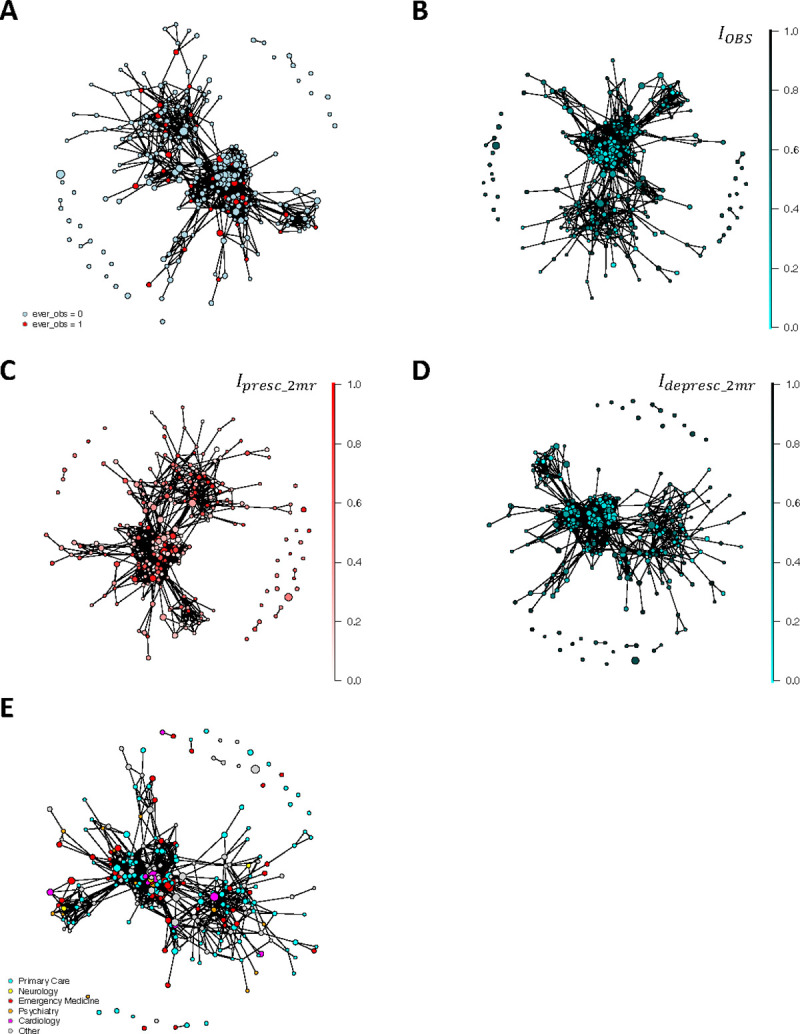
Egocentric network of the physician with maximum node degree (N = 276) in the LCC of the prescribing network. The ego physician was removed from the plot for the clarity of presentation. The ties shown in the plots are among the peers of the ego physician. The nodes are sized by physician annual volume (the number of distinct patients treated throughout the year). The colors of nodes correspond to their prescribing behavior or specialties. A) The connections among physicians are distinguished by whether they have ever contributed to bringing patients to the riskiest prescription state (the OBS state). B) The connections among physicians where the node color represents the value of $I_{OBS}$, the proportion of times they bring their patients to prescription state OBS. C) The connections among physicians where the node color represents $I_{\mathrm{pres}c_{2}mr}$, the proportion of prescribing events when two or more drugs are prescribed at once to the patients. D) The connections among physicians where the node color represents $I_{\mathrm{depres}c_{2}\mathrm{mr}}$, the proportion of deprescribing events at which two or more drugs are deprescribed at once to the patients. E) The connections among physicians are colored by their specialties.

Figure 5 shows the homophily patterns in terms of prescribing and deprescribing in the ego network. An important observation is that central physicians with higher patient volume and higher node degrees have lower risky prescribing intensity than peripheral physicians. In addition, there are closely positioned clusters of physicians with similar prescribing intensity and behavior.

On the HRR-level (Table 4), 6 out of 12 HRRs show significant homophily in terms of the index $I_{OBS}$ of riskiest prescribing, and 10 of them show significant homophily in terms of the index quantifying the intensity of the addition of drugs in two of the three drug classes, $I_{\text{presc}2mr}$. The level of homophily varies across the HRR levels. The signs of the estimated coefficients were almost exclusively negative, implying that the more similar the measure the more likely a tie is to be present (positive homophily). For other prescribing or deprescribing indexes, we do not see as significant nor prevalent homophily as at the state level, which is consistent with these transitions being less common and thus having less information to estimate their coefficients. The discrepancy of prescribing-associated homophily between the state and HRRs, especially the homophily found at the state-level but not in some of the HRRs, may indicate that some prescribing groups at the state-level rely on cross-HRR physician patient-sharing.

### 4.4 Triadic-level hyper homophily

**Fig. 6:**
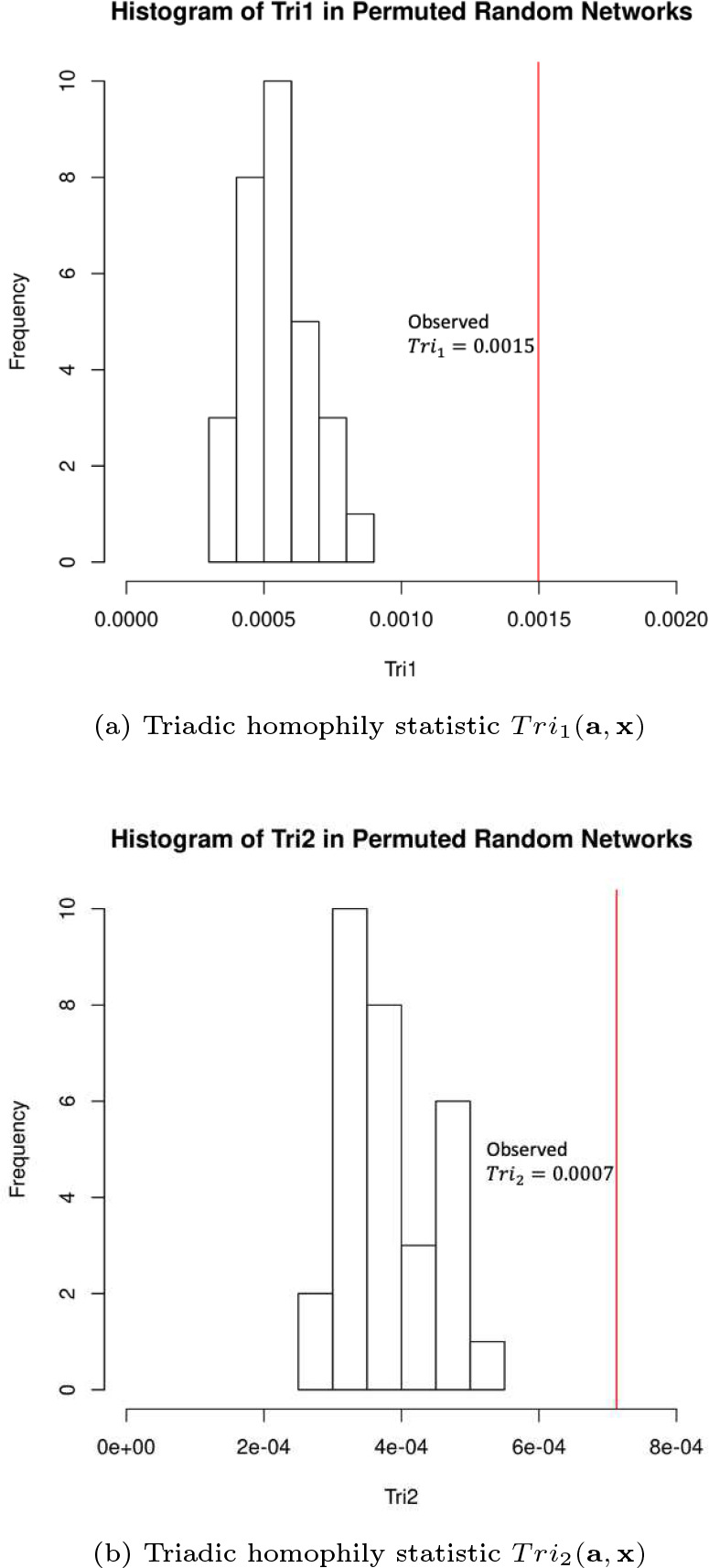
Histogram of triadic homophily network statistics generated by the non-parametric test for triadic homophily. The triadic homophily statistic ${Tri}_{1}(a,x)$ is the proportion of closed triangles in the network in which each node has the $I_{\mathrm{ever}OBS}\;$ node attribute, reflecting whether a physician has ever contributed to bringing patients to the riskiest prescription state OBS. The triadic homophily statistic ${Tri}_{2}(a,x)$ is the proportion of open two-paths with all nodes having the same attribute that are closed in the network. Panel (a) is the histogram of ${Tri}_{1}(a,x)$ and panel (b) is the histogram of ${Tri}_{2}(a,x)$ calculated from 30 networks with randomly shuffled node attributes under the null hypothesis of no homophily with respect to the given prescribing index. The red vertical lines denote the values in the observed network.

The realized values of the triad-level risky prescribing index quantified by the network statistics ${Tri}_{1}(a,\;x)$ and ${Tri}_{2}(a,\;x)$ are 0.0015 and 0.0007, respectively (Figure 6). The interpretation of the realized ${Tri}_{1}(a,\;x)$ is that among 10,000 closed triangles (mutual patient-sharing within all three physician dyads), 15 of them include nodes with the same attribute (the three physicians each contributed to bringing at least one patient to the riskiest prescription state OBS). The interpretation of ${Tri}_{2}(a,x)$ is that among 10,000 open two-paths (2-stars) with the same node attribute $\left(I_{\mathrm{everOBS}}\right)$ in the network, 7 of them are closed. The attribution re-distribution permutation test found that the observed values of ${Tri}_{1}(a,x)$ and ${Tri}_{2}(a,x)$ in the network were significantly higher than expected (p=0.000). These results suggest that 1) when three physicians share patients among themselves, they are all more likely to be all involved in risky prescribing than by chance; and 2) when two physicians share patients with a common third physician, and all three have been involved in risky prescribing, then these two physicians are more likely to also share patients between them than by chance. These results suggest that risky prescribing is driven by a higher-order form of homophily representing a more complex network phenomena than traditional dyadic homophily.

## 5 Conclusions

This paper has made several methodological contributions to quantify physicians’ prescribing and deprescribing behaviors comprehensively and to study homophily associated with prescribing in a shared-patient physician network. Heuristic algorithms were designed to attribute a deprescribing event to a responsible physician. We represented patients’ drug exposures as prescription states and modeled patients’ transitions between these prescription states. Physicians’ contributions to prescribing and deprescribing were quantified through their contribution to patients’ prescription state transitions with various measures being derived from the physician responsibility transition matrices. This quantitative framework can be generalized to comprehensively quantify any other prescribing behaviors by not only incorporating the number and classes of patients’ drug exposures but also the changes in the drug exposures reflected by the time sequences of prescribing and deprescribing events.

Another methodological contribution is the development of two triadic homophily network statistics and associated statistical tests that avoided degeneracy in ERGMs including the *triangle* and other triadic statistics enumerated over the network. These two network statistics advance the study of homophily from dyads to triads. Although we use standalone non-parametric random redistribution (partial permutation) tests to compare the observed statistics to those expected by chance, such statistics may be incorporated in ERGMs as network statistics so that other network statistics can be simultaneously controlled. Another direction of future research is to develop methods of generating the null distribution of the triadic homophilly statistics considered herein while preserving the distribution of network statistics beyond density and reciprocity; for example, the preservation of the entire degree distribution.

In the risky prescribing application, we discovered substantial homophily of prescribing behaviors among physicians, as well as the assortative and disassortative mixing patterns with respect to physician specialty in the prescribing network. We also found significant risky-prescribing-associated homophily at the triadic level in the observed network compared to that expected by chance. We found that physicians’ level of involvement in prescribing and deprescribing varied across specialties, and variation in prescribing-associated homophily across HRRs. Our findings of the homophily associated with prescribing behaviors and physicians’ specialties in the shared-patient physician network provide a basis for promoting guideline-concordant prescribing practice and informing interventions (Ran et al, 2024). The act of sharing patients can be a channel for behavior changes and so physicians who only share patients with risky prescribers might expose the focal physician to so much high-risk behavior that their own practice changes, forming a loop of problematic prescribing. Given this potential reinforcement of influence between physicians when homophily exists (Centola, 2011), external interventions may be warranted to help break the cycle of risky prescribing among communities of guideline non-concordant prescribers. The substantial variation in prescribing-associated homophily across HRRs revealed by our HRR-stratified models reinforces previous literature on the geographic variability in physician patient-sharing network characteristics (Landon et al, 2012).

Understanding the assortative and disassortative patterns among different physicians’ specialties in the context of prescribing may provide an overarching view of the patient flow between different types of providers when patients seek medical care and prescriptions. The finding that prescribers in psychiatry were more likely to share patients with each other may be due to the complexity of conditions they generally encounter, requiring their patients to have multiple visits to different psychiatrists. In contrast, PCPs often refer patients to secondary care and are less likely to share patients among themselves. Our findings also suggest variations in prescribing intensity across different physician specialties may help target interventions to address risky prescribing. For example, if policymakers were to impose guidelines to promote safe prescribing among different physician specialties, they may expect differential impacts as specialties do not all have the same baseline prescribing intensities and some of the specialties (e.g., psychiatry) may have better outcomes of interventions because they are more likely to share patients with physicians in the same specialty than across specialties.

This study is subject to several limitations. First, although our study is the first to our knowledge to deduce deprescribing from administrative claims data, not having deprescribing events recorded let alone time-stamped in claims data meant that we needed to define deprescribing heuristically, resulting in the attribution of deprescribing not being as sensitive as ideal (as evinced by the distributions of $I_{0}$ and $I_{1}$ being dominated by prescribing events). The availability of Electronic Health Record (EHR) data might have enabled deprescribing to be more accurately identified. However, even given the limited data we had, we found that the involvement of deprescribing in our indexes was helpful. Second, the data used in this study was cross-sectional, which led to challenges in estimating network effects beyond homophily effects. The availability of longitudinal data would have allowed dyadic dependent network effects to be modeled as lagged variables to avoid the problem of degeneracy (Paul and OḾalley, 2013). Longitudinal data would also have allowed the process of social selection (i.e., the factors governing the selection of relationships (Runciman et al, 2009)) to be distinguished from social influence (i.e., the process of one individual exerting influence on another so that they adopt similar traits). Third, our study focused on the Medicare population, whereas the same research question within younger populations is also of interest.

In summary, we have proposed a novel framework to model the relationship between informal physician professional networks and new measures for quantifying physicians’ prescribing and deprescribing behavior. We discovered significant homophily associated with prescribing among physicians’ connections through sharing patients. These findings provide important insights into the mechanism underlying the spread of risky prescribing among the older population in the United States and of how communities of prescribers emerge and evolve. We hope that this work helps to incentivize interventions to reduce practices that are not compliant with guidelines and to promote safe practices among healthcare providers.

## Figures and Tables

**Table 1:** Definitions of ERGM terms for undirected networks and interpretations

Terms	Math definition	Interpretation

edges	$m=\frac{1}{2}\sum_{ij}a_{ij}$	Number of edges in the network; controls for network density

Node attribute		
nodefactor	$Z_{k}=\frac{1}{2}\sum_{ij}\left(1\left(x_{i}=k\right)+1\left(x_{j}=k\right)\right)a_{ij}$	Number of times a node possessing a categorical attribute of value k appears on an edge in the network
nodecov	$Z=\frac{1}{2}\sum_{ij}\left(x_{i}+x_{j}\right)a_{ij}$	For continuous attributes, the sum of the attribute across node pairs for all edges present in network

Homophily term		
nodematch	$S=\frac{1}{2}\sum_{l}\sum_{ij}\left(1\left(x_{i}=l\right)1\left(x_{j}=l\right)\right)a_{ij}$	Uniform homophily; the number of edges when two nodes have the same categorical attribute
nodematch	$S_{l}=\frac{1}{2}\sum_{ij}\left(1\left(x_{i}=l\right)1\left(x_{j}=l\right)\right)a_{ij}$	Differential homophily; the number of edges when two nodes have the same categorical attribute taking a value of l
absdiff	$S=\frac{1}{2}\sum_{ij}\left|x_{i}-x_{j}\right|a_{ij}$	For continuous attributes, the sum of absolute differences in the attribute within a dyad across all edges present in the network

Note: $A\;=\;[a_{\mathrm{ij}}]$ is the adjacency matrix of the binary-undirected network in this study, and $a_{\mathrm{ij}}$ = 1 if physician i and j shared patients during 2014. The variables $x_{i}$ and $x_{j}$ are the node attributes of physician i and j, and k and l denote the values of a categorical attribute held by the two actors comprising a dyad.

**Table 2:** Network statistics of the largest connected component of the shared-patient physician prescribing network (specific to prescriptions of opioids, benzodiazepines, and sedative-hypnotics) for Ohio in 2014.

	Shared-patient physician network		

	Whole net	Prescribing net	LCC of prescribing net

**Network statistics**			
Number of nodes	35765	22655	17363
Number of ties	494462	265112	261816
Density	0.0008	0.0010	0.0010
Number of components	3002	2056	1
Size of LCC	27503	17363	17363
Degree (mean, IQR, SD)	27.7 (n/a, 44.0) (37.5)	23.4 (n/a, 38.0) (28.7)	30.2 (n/a, 45.0) (29.6)
Global clustering	0.168	0.171	0.171
Average path length	4.663	4.599	4.599
**Prescribing statistics**			
*I*_0_ (mean, IQR)		0.871 (0.843, 1.0)	0.876 (0.875, 1.0)
$I_{OBS}$ (mean, IQR)		0.009 (0.0, 0.0)	0.009 (0.0, 0.0)
$I_{\mathrm{everOBS}}$ (# of 1, # of 0)		(1972, 20683)	(1412, 15951)
$I_{\mathrm{presc2mr}}$ (mean, IQR)		0.030 (0.0, 0.0)	0.029 (0.0, 0.0)
$I_{\mathrm{depresc2mr}}$ (mean, IQR)		0.017 (0.0, 0.0)	0.016 (0.0, 0.0)
Volume (mean, IQR, SD)	62.7 (n/a, 96.0) (86.3)	70.6 (n/a, 112.0) (87.8)	91.6 (18.0, 136.0) (90.3)

Note: The physician network is constructed based on the overlap of patient care at any point during 2014 between physician pairs treating patients residing in Ohio. The prescribing network is a subset of the whole network where its physicians have prescribed at least one opioid, benzodiazepine, or sedative-hypnotic during 2014. Volume is the number of Ohio Medicare fee-for-service beneficiaries a physician encountered throughout 2014. The entries of n/a signify that the true value is suppressed to satisfy data suppression rules designed to protect patient privacy by the Center for Medicare and Medicaid Services. LCC = largest connected component.

**Table 3:** ERGM adjusted homophily effects for models estimated on the largest connected component of the Ohio 2014 shared-patient physician prescribing network.

	Model 1			Model 2			Model 3			Model 4		

	Est.	SE	p	Est	SE	p	Est	SE	p	Est	SE	p

Edges	−5.732	0.012	***	−5.614	0.006	***	−5.609	0.006	***	−5.620	0.006	***
**Node attribute Prescribing**												
***Binary***												
$I_{\mathrm{everOBS}}$ = 1	0.327	0.010	***									
***Continuous***												
$I_{OBS}$				1.202	0.109	***						
$I_{\mathrm{presc2mr}}$							0.492	0.049	***			
$I_{\mathrm{depresc2mr}}$										0.337	0.066	***
**Specialty (ref. PC)**												
EM	−0.584	0.006	***	−0.614	0.006	***	−0.614	0.006	***	−0.611	0.006	***
Neuro	−0.338	0.010	***	−0.358	0.010	***	−0.359	0.010	***	−0.357	0.010	***
Psych	−0.668	0.009	***	−0.654	0.009	***	−0.653	0.009	***	−0.651	0.009	***
Other	−0.396	0.004	***	−0.423	0.005	***	−0.423	0.005	***	−0.420	0.005	***
**Prescribing homophily**												
***Binary***												
$I_{\mathrm{everOBS}}$ = 1	0.037	0.011	***									
***Continuous***												
absdiff($I_{OBS}$)				−1.200	0.114	***						
absdiff($I_{\mathrm{presc2mr}}$)							−0.619	0.054	***			
absdiff($I_{\mathrm{depresc2mr}}$)										−0.203	0.068	**
**Specialty homophily**												
PC	−1.509	0.009	***	−1.509	0.009	***	−1.511	0.009	***	−1.509	0.009	***
EM	0.541	0.019	***	0.541	0.019	***	0.541	0.019	***	0.541	0.019	***
Neuro	−0.190	0.091	*	−0.190	0.091	*	−0.190	0.091	*	−0.190	0.091	*
Psych	0.673	0.050	***	0.670	0.050	***	0.674	0.050	***	0.673	0.050	***

Note: The node attribute term and homophily term for an attribute were added one at a time in the model for each of the prescribing or deprescribing indexes, yielding five separate models. In each of the models, physician specialty and homophily of physician specialty (restricted to uniform effects across the different specialties) were included in the model. Absdiff is the ERGM term for examining the homophily of a continuous node attribute, with a negative estimate indicating homophily (smaller differences imply a higher likelihood of a network connection). Abbreviations: PC = primary care, EM = emergency medicine, Neuro = neurology, Psych = psychology. Significance levels:

*** *p* < 0.001

** *p* < 0.01

* *p* < 0.05.

**Table 4:** ERGM adjusted homophily effects in HRR shared-patient sub-networks in 2014.

Homophily effects of indexes										

Descriptive Stats			absdiff($I_{OBS}$)		absdiff($I_{\mathrm{presc2mr}}$)		absdiff($I_{\mathrm{depresc2mr}}$)		$I_{\mathrm{everOBS}}$	

HRR	N	Density	Coef.	SE	Coef.	SE	Coef.	SE	Coef.	SE

180	129	0.116	−3.998	3.662	0.004	1.623	1.052	1.898	0.026	0.133
357	193	0.106	0.810	2.707	−1.541*	0.714	0.467	0.775	−0.268	0.149
331	256	0.128	−5.156	2.675	−1.792*	0.697	−0.325	0.773	0.047	0.117
332	415	0.048	−1.765*	0.745	−0.863***	0.250	0.299	2.410	0.056	0.079
335	550	0.060	−1.887*	0.920	−0.881**	0.305	−0.545	0.416	0.063	0.070
326	648	0.050	−1.281	0.795	−1.066***	0.306	44.210	280.321	−0.080	0.056
325	750	0.030	−0.469	0.814	−0.917**	0.279	0.496	0.699	0.0002	0.089
334	1039	0.029	−0.532	0.416	−1.190***	0.226	−0.301	0.209	0.018	0.045
330	1164	0.024	−1.205**	0.419	−0.339	0.196	−0.071	0.319	0.0002	0.037
327	1760	0.015	−1.711**	0.584	−0.783***	0.179	−0.362*	0.171	0.120**	0.044
328	2623	0.010	−1.603***	0.370	−0.897***	0.142	−0.193	0.227	0.060	0.034
329	3101	0.008	−1.181***	0.234	−0.754***	0.109	−0.327*	0.157	−0.018	0.025

Note: The HRR sub-networks were partitioned from the largest connected component of the Ohio 2014 shared-patient physician prescribing network. These sub-networks were not restricted to their respective largest connected components; thus, they may not be fully connected. Significance levels:

*** *p* < 0.001

** *p* < 0.01

* *p* < 0.05.

## Data Availability

The data used for the motivating analyses contains patient-identifiable information and so cannot be made available. However, R code for performing simulation studies (which can be easily adapted to analyze a real data set) is available from the paper’s GitHub site: https://github.com/xinran02/PolyRxNetworkHomophily

## Appendix

**Algorithm 1:** Pseudo code: Attributing the physicians responsible for deprescribing prescriptions

**Input:** A set of n prescriptions patient h receives and their prescribing physicians (a lone physician responsible for deprescribing prescription s is denoted $P_{\mathrm{hs}}$).	
**Output:** The responsible physician $P_{\mathrm{hs}}$ for deprescribing the prescription of interest.	
1:	**for** *s* = 1, 2, ..., *n* **do**
2:	**if** length of prescription *s* >= 30 days **then**
3:	Δ ← empty list ▷ difference between two dates
4:	**for** *w* = *s* + 1, *s* + 2, ..., *n* **do**
5:	$b_{w}$ ← begin date of prescription w
6:	$e_{s}$ ← end date of prescription s
7:	**if** $e_{s}\;-\;b_{w}$ > 0 and $e_{s}\;-\;b_{w}$ <= 30 **then** ▷ prescription s discontinues within 30 days of encountering a physician
8:	Add $e_{s}\;-\;b_{w}$ to Δ
9:	**end if**
10:	**end for**
11:	Find the prescription p with minimum Δ and corresponding prescriber $P_{\mathrm{hp}}$ ▷ most recent encountered physician
12:	responsible physician for deprescribing prescription p ← physician $P_{\mathrm{hp}}$
13:	**if** multiple responsible physicians **then**
14:	contribution weight ← 1 / number of contributors
15:	**end if**
16:	**if** no physician is held accountable after applying above criteria **then**
17:	responsible physician for deprescribing prescription p ← a pseudo-physician
18:	**end if**
19:	**else**
20:	responsible physician for deprescribing prescription s ← a pseudo-physician
21:	s←s+1
22:	**end if**
23:	**end for**

**Algorithm 2:** Pseudo code: Determining the deprescribing state transition to attribute to the responsible physicians

**Input:** (1) The responsible physician $P_{\mathrm{hs}}$ for deprescribing at prescription transition s for patient h; (2) a set of n prescriptions patient h receives.	
**Output:** The deprescribing state transition that physician k is responsible for.	
1:	$D_{b}$ ← empty list ▷ List of drugs patient h is taking before discontinuation of prescription s
2:	$D_{a}$ ← empty list ▷ List of drugs patient h is taking after discontinuation of prescription s
3:	**for** *w* = 1, 2, ..., *n* **do**
4:	$b_{w}$ ← begin date of prescription w
5:	$e_{w}$ ← end date of prescription w
6:	$e_{s}$ ← end date of prescription s
7:	**if** $e_{s}\;–\;0.1\;>\;s_{w}$ and $e_{s}\;–\;0.1\;<\;e_{w}$ **then**
8:	Add w to $D_{b}$
9:	**end if**
10:	**if** $e_{s}\;+\;0.1\;>\;s_{w}$ and $e_{s}\;+\;0.1\;<\;e_{w}$ **then**
11:	Add w to $D_{a}$
12:	**end if**
13:	**end for**
14:	determine prescription state u based on $D_{b}$
15:	determine prescription state v based on $D_{a}$
16:	physician k is responsible for patient *h*’s deprescribing state transition from u to v
